# Isolated posterior ST-elevation myocardial infarction: the necessity of routine 15-lead electrocardiography: a case series

**DOI:** 10.1186/s13256-022-03570-w

**Published:** 2022-08-28

**Authors:** Mochamad Yusuf Alsagaff, Rizki Amalia, Budi Baktijasa Dharmadjati, Yolande Appelman

**Affiliations:** 1 Department of Cardiology and Vascular Medicine, Faculty of Medicine, Airlangga University-Dr Soetomo General Hospital Surabaya, Jalan Mayjen Prof. Dr. Moestopo No. 6-8, Surabaya, 60286 Indonesia; 2 Department of Cardiology, VUmc-University of Amsterdam, Amsterdam, North Holland The Netherlands

**Keywords:** Isolated posterior myocardial infarction, ECG, Posterior leads, 15-Lead ECG, Reperfusion therapy

## Abstract

**Background:**

True isolated posterior myocardial infarction is an uncommon finding of acute coronary syndrome, with an incidence rate of 3–7%. The prevalence rates of isolated posterior myocardial infarction in men and women are 72% and 28%, respectively. This uncommon finding may be attributed to multiple factors, such as unremarkable changes on 12-lead electrocardiography, a lack of awareness or knowledge, and an absence of diagnostic consensus, which leads to reperfusion delay and poor clinical outcomes.

**Case summary:**

Herein, we report three cases of acute myocardial infarction presenting as isolated ST-segment elevation in the posterior leads (V7–V9): Asian men aged 57, 62, and 53 years, who presented with ST-segment depression in V1–V3 that resolved gradually. Coronary angiography revealed a total/critical occlusion of the proximal circumflex coronary artery in all three cases. Routine and accurate interpretations of 15-lead electrocardiography (12-lead with additional V7–V9) resulted in a better sensitivity for isolated posterior myocardial infarction diagnoses, followed by a timely and opportune primary percutaneous coronary intervention.

**Conclusions:**

Isolated posterior myocardial infarction is a rare but potentially fatal event that is often accompanied by atypical and subtle changes on 12-lead electrocardiography (especially in the V1–V3 precordial leads) and may remain undetected by physicians. Therefore, the comprehensive and routine application of posterior leads is a crucial addition to the standard diagnosis and management of acute coronary syndrome in patients with subtle ST-segment changes who do not fulfill the criteria for ST-elevation myocardial infarction.

## Background

True isolated posterior myocardial infarction (IPMI) is a rare condition. However, it may be one of the most commonly undiagnosed types of acute myocardial infarction (MI) owing to its low incidence; unremarkable findings on standard 12-lead electrocardiography (ECG); and a lack of awareness, knowledge, and consensus regarding its diagnostic criteria [1]. The clinical presentation of IPMI is not different from that of other MIs. However, the absence of typical changes on the standard 12-lead ECG pattern (such as ST-segment elevation) can lead to a delay in the performance of primary percutaneous coronary intervention (PPCI) [2].

The term IPMI is used to indicate the occurrence of necrosis in the dorsal infra-atrial region of the left ventricle, which is located under the left atrioventricular sulcus [3]. A majority of patients with IPMI have stenosis or occlusion of the left circumflex coronary artery (LCx) [4]. The LCx is the dominant vessel in 15% of patients, supplying the left posterior descending artery from the distal continuation of the LCx. It provides blood supply to the posterior part of the left ventricle [5].

Acute MI, which involves the posterior wall of the left ventricle, accounts for 15–20% of the total cases of acute MI [1]. In routine practice, a 12-lead ECG is used to diagnose ST-elevation myocardial infarction (STEMI), especially for the anterior [left anterior descending artery (LAD) territory] and inferior [right coronary artery (RCA) territory] walls of the left ventricle. Meanwhile, additional ECG leads (V7–V9) reflect the activity of the posterior wall of the left ventricle. These additional leads have increased the rate of IPMI diagnoses from “very rare” to 3.3% among all patients with acute MI [2, 6].

On standard 12-lead ECG, the typical indication for MI (ST-segment elevation) of the posterior wall appears as ST-segment depression on the precordial side because the posterior endocardial wall is opposite to the anterior endocardial wall. Specifically, if there is an ST-segment deviation (horizontal > downslope/upslope) that is accompanied by a prominent R wave (R/S > 1 in lead V2) and prominent T waves (or a combination of a deviated horizontal ST-segment and prominent T waves) in leads V1–V3, then it will appear as an ST-segment elevation or Q wave caused by acute IPMI when the ECG is reversed [7].

One of the major goals of the emergency department is to identify patients who may benefit from thrombolysis or a PPCI strategy as soon as possible [8]. This case series identified some of the subtle findings on 12-lead ECG that are suggestive of IPMI. Our case series aimed to demonstrate the usefulness of additional leads in identifying ST-segment elevation in the setting of IPMI. Awareness and knowledge regarding this phenomenon will lead to better treatment and correspondingly higher survival rates in patients with IPMI.

## Case presentation

**Fig. 1:**
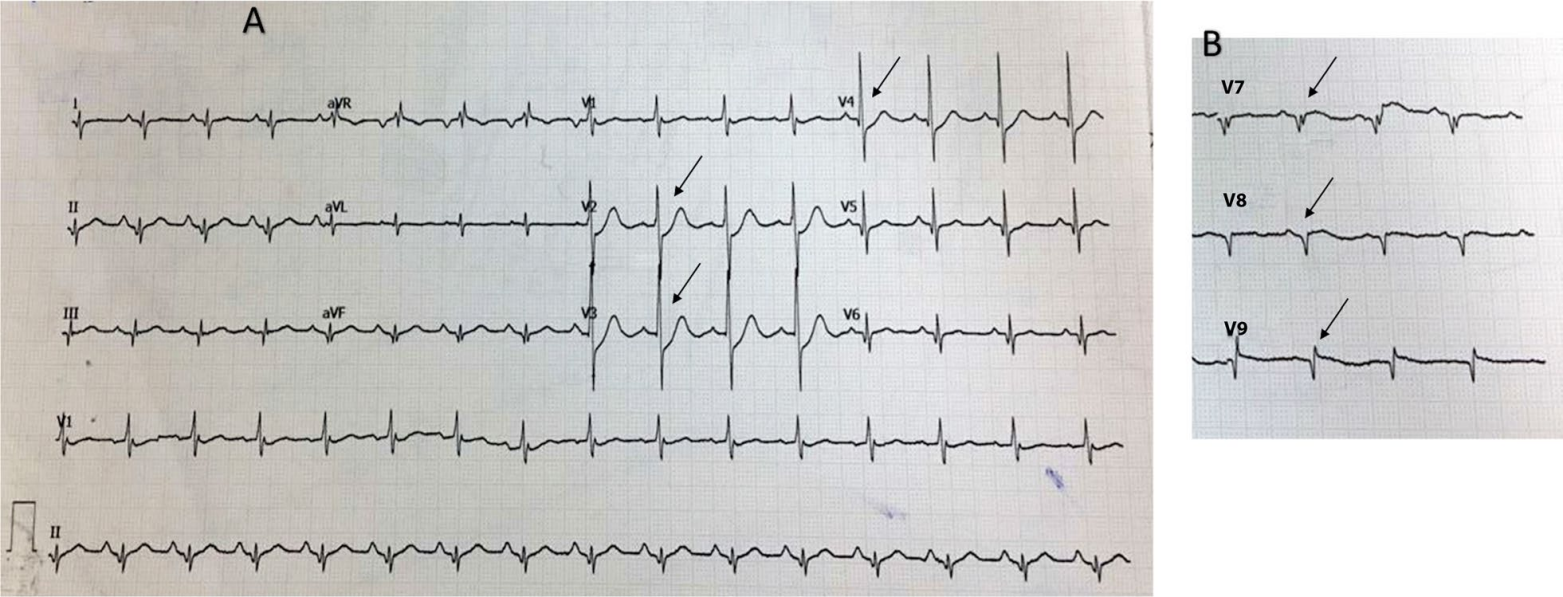
Referring hospital 12-lead ECG. **A** Arrows display anteroseptal (V2–V4) upsloping ST-segment depression. **B** Arrows display posterior ST elevation in the additional V7–V9 leads

**Fig. 2:**
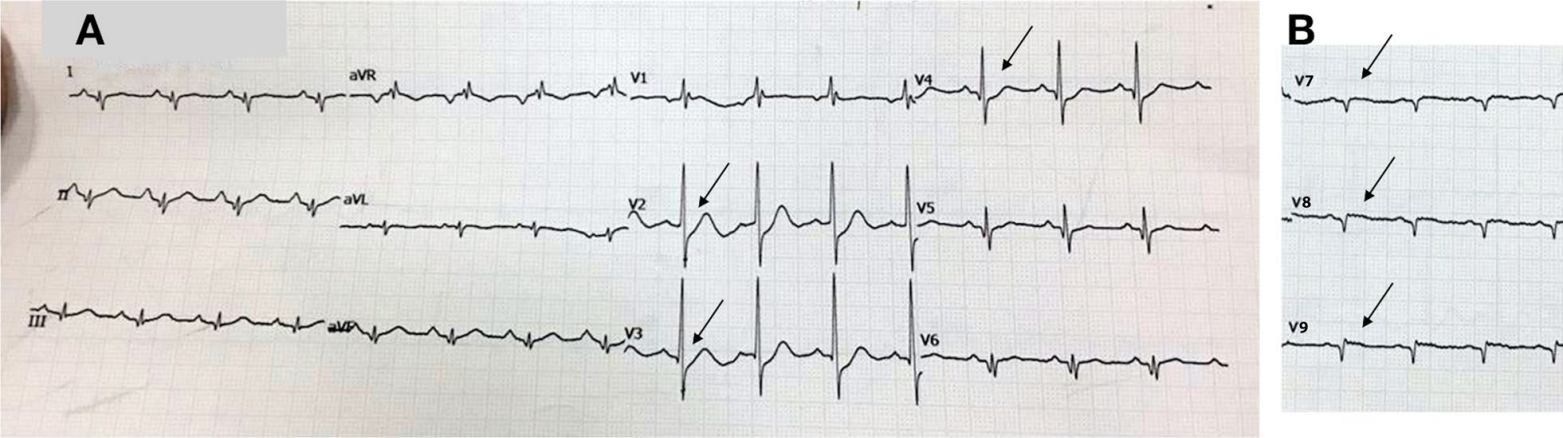
The 12-lead ECG upon arrival in the PPCI center. **A** Arrows display gradually resolving ST-segment depression in the anteroseptal leads (V2–V4). **B** Arrows display persistent ST elevation in the additional V7–V9 leads

The first case included a 57-year-old Asian man, who was referred to our hospital with typical chest pain for the past 2 days, along with dyspnea. He had hypertension and was a smoker, but was not diabetic and did not have any previous history of heart disease. His vital signs were as follows: blood pressure, 105/70 mmHg; pulse rate, 95 beats per minute; respiration, 25 breaths per minute; and peripheral capillary oxygen saturation, 96%. Lung examination revealed minimal bibasilar rales. The ECG conducted at the referring hospital showed upsloping ST depression in V2–V4 and ST elevation in V7–V9 (Fig. 1). Serial ECG examination at our hospital showed a gradual resolution of ST depression in the anteroseptal region, despite persistent ST elevation in the posterior lead (Fig. 2). Additionally, the troponin T value increased to 5413 ng/L. Echocardiography revealed a mild hypokinetic posterior wall with a left ventricular ejection fraction of 58%.

**Fig. 3:**
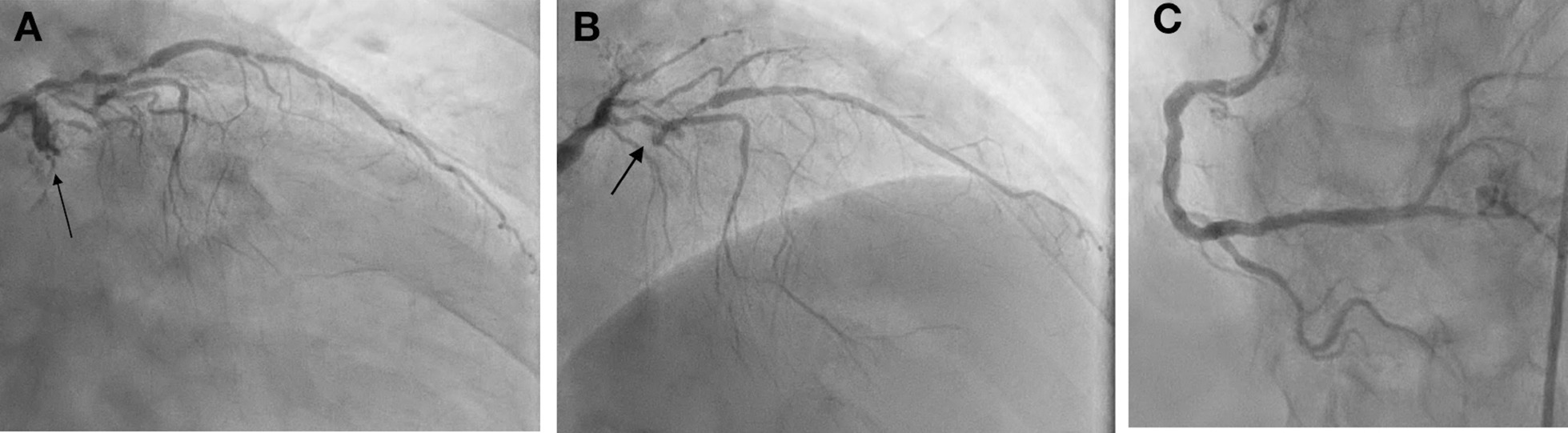
Coronary angiogram that was performed at the PPCI center, which shows total occlusion of the proximal LCx (right anterior oblique caudal view, arrow in **A**), chronic total occlusion of the proximal LAD (anteroposterior cranial view, arrow in **B**), and diffusely diseased RCA (left anterior oblique view, **C**)

**Fig. 4:**
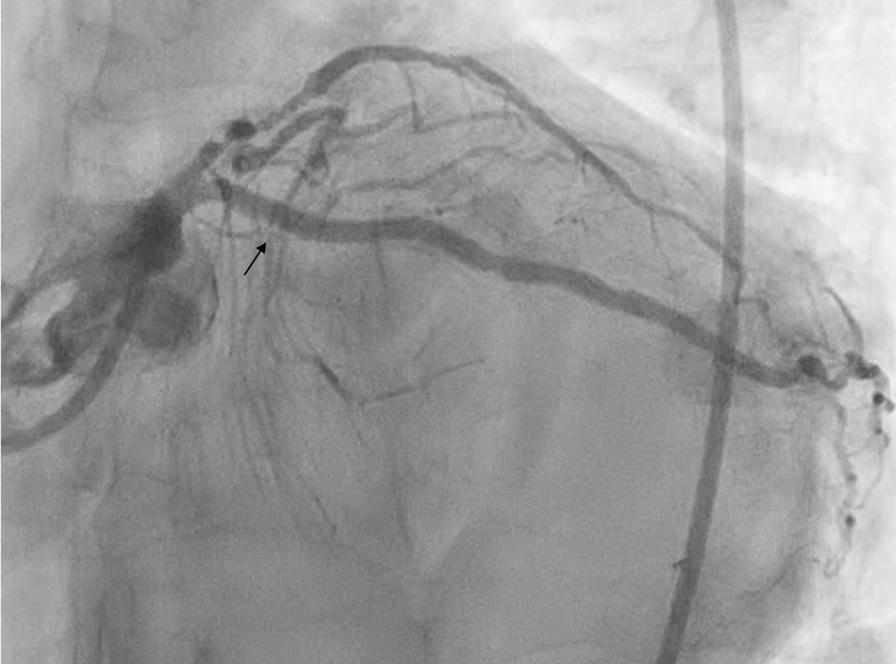
Left anterior oblique caudal view of left coronary angiogram after DES implantation in proximal LCx, TIMI 3 flow (arrow)

**Fig. 5:**
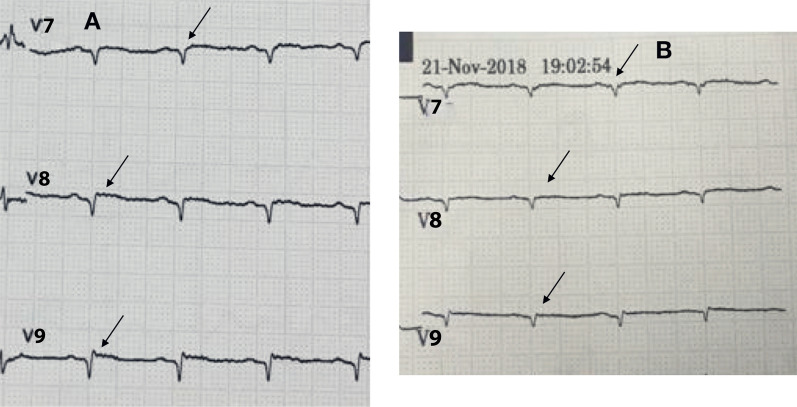
**A** The posterior lead ECG before PPCI; arrows display ST-segment elevation in V7–V9. **B** The posterior leads ECG at the PPCI center post-revascularization; arrows display ST-segment resolution in V7–V9

A posterior STEMI Killip class II was confirmed. Coronary angiography revealed total occlusion of the proximal LCx, chronic total occlusion in the proximal left anterior descending (LAD), and a diffusely diseased right coronary artery (RCA) (Fig. 3). Proximal LCx, including the proximal–distal obtuse marginal, was treated with a drug-eluting stent. After the procedure, The Thrombolysis in Myocardial Infarction Score (TIMI 3) flow was observed in the LCx (Fig. 4) and ST-segment resolution was seen on ECG (Fig. 5).

**Fig. 6:**
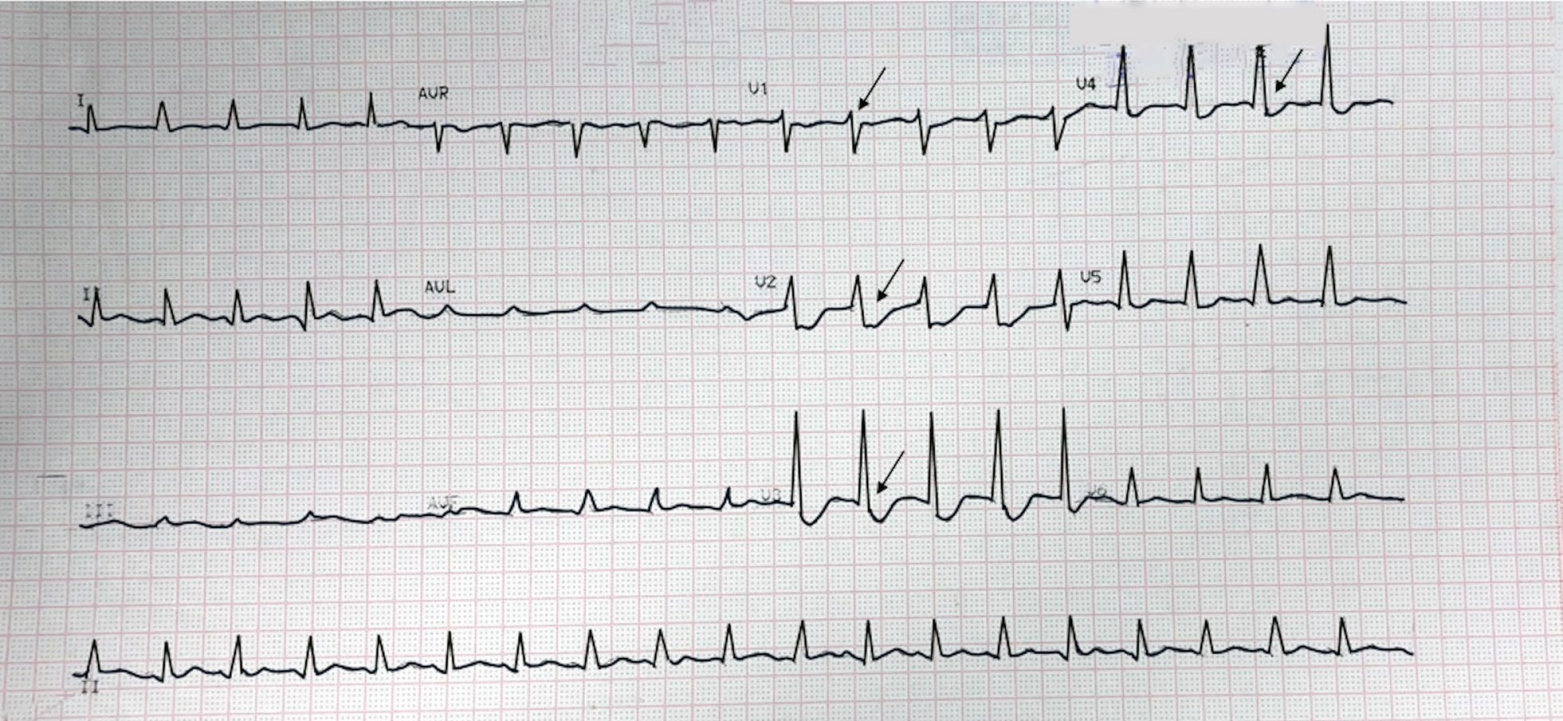
ECG of the referring hospital; arrows display anteroseptal ST-segment depression (V1–V4)

**Fig. 7:**
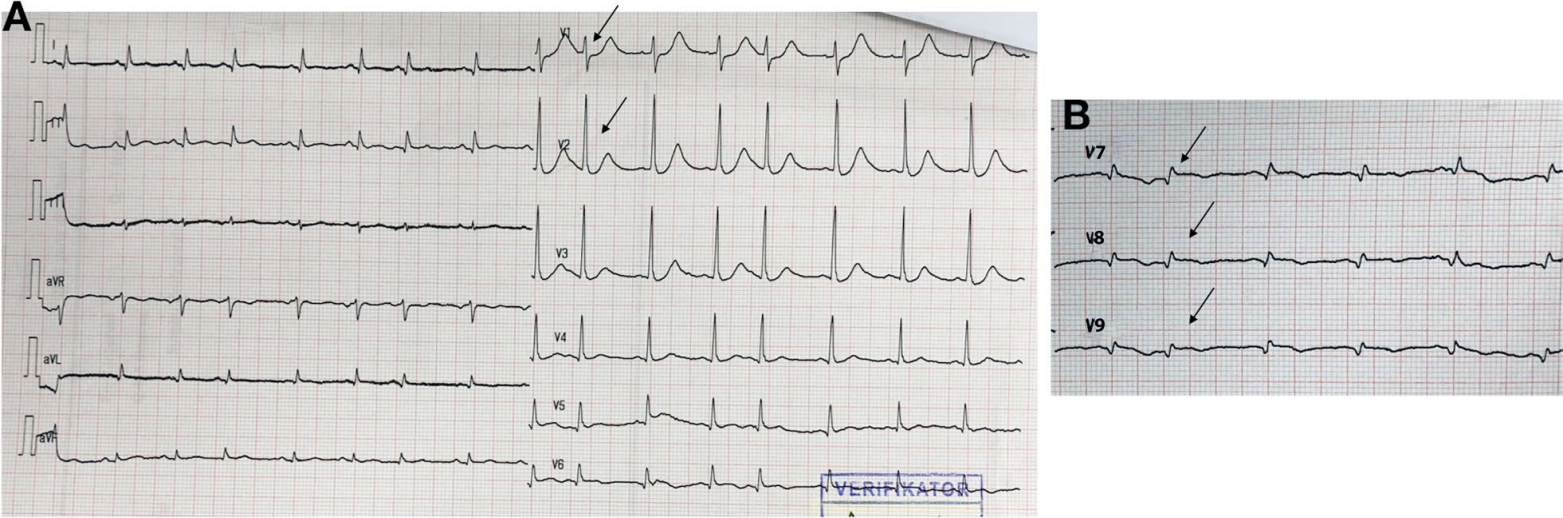
The ECG that was performed upon arrival at the PPCI center. **A** Arrows display 0.5 mm ST-segment depression in V1–V3. **B** Arrows display posterior ST-segment elevation in V7–V9

The second case included a 62-year-old Asian man diagnosed with non-ST-elevation myocardial infarct (NSTEMI) and referred to our hospital with an episode of ventricular fibrillation (VF) for which he was defibrillated once. He complained of typical chest pain for more than 20 hours. He had a history of smoking but was not hypertensive or diabetic, and did not have previously diagnosed heart disease. Upon admission to our hospital, his vital signs showed a blood pressure of 90/60 mmHg with norepinephrine and dobutamine support, pulse rate of 100 beats per minute, respiratory rate of 20 breaths per minute, and peripheral capillary oxygen saturation of 98%. Chest examination showed no abnormalities. The ECG at the referring hospital showed a > 3 mm horizontal ST-segment depression in V1–V4 (Fig. 6), but a posterior lead ECG (V7–V9) was not performed. However, the ECG in our hospital (90-minute difference from the first ECG) revealed a 0.5-mm ST-segment depression in V1–V3 with an ST-segment elevation in V7–V9 (Fig. 7). Moreover, the troponin T value increased to 4068 ng/L. Echocardiography revealed a hypokinetic posterior, anteroseptal, and lateral wall, with a left ventricular ejection fraction of 49%.

**Fig. 8:**
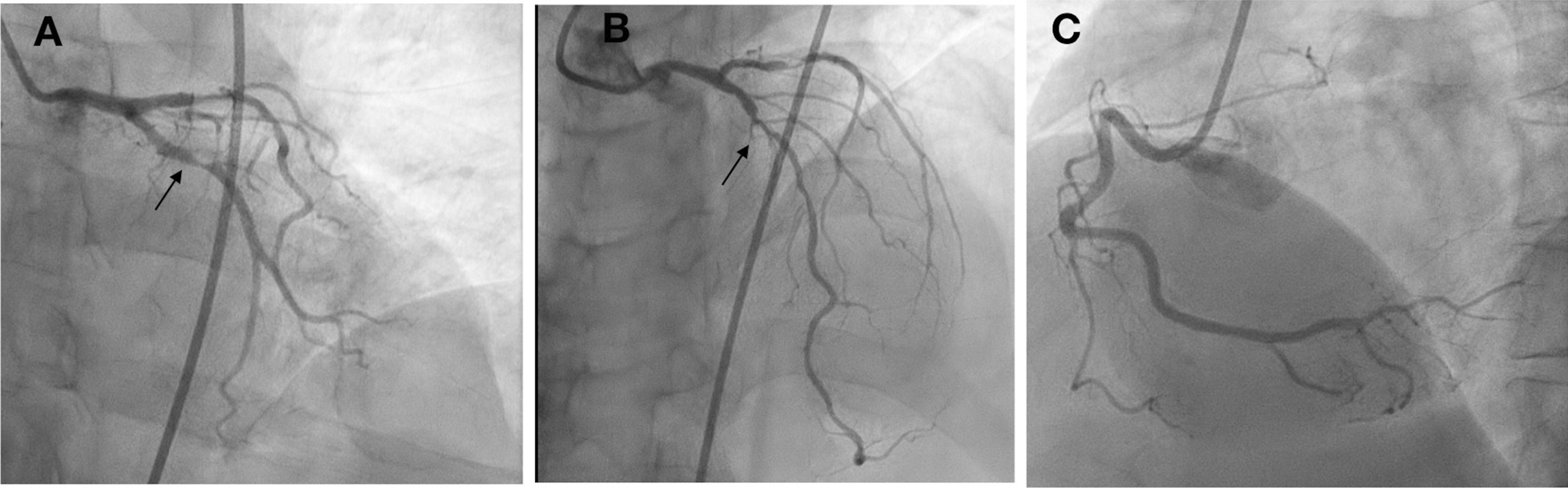
Coronary angiography showing 98% stenosis in the proximal LCx (anteroposterior caudal view, arrow in **A**), 80% stenosis in the proximal LAD (anteroposterior cranial view, arrow in **B**), and normal RCA (anteroposterior cranial view, **C**)

**Fig. 9:**
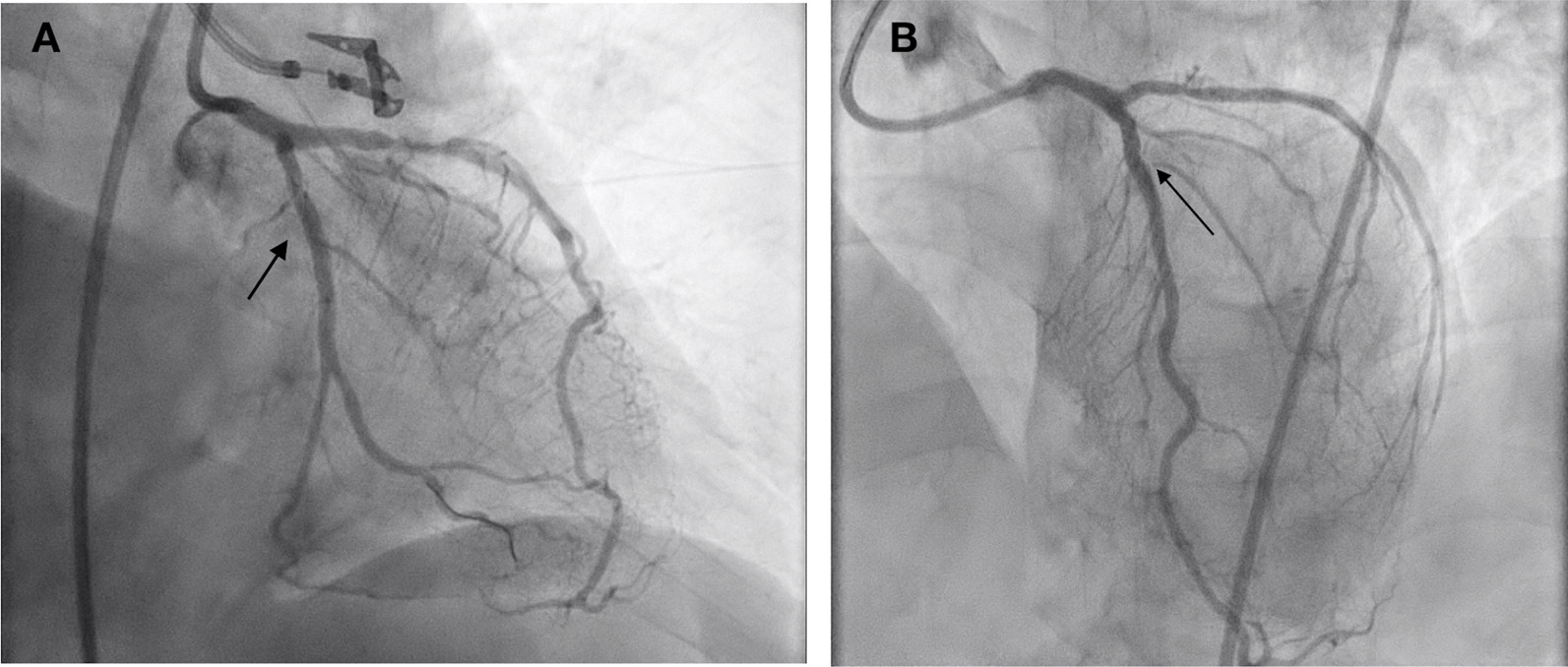
Post-PCI procedure: proximal LCx DES implantation (right anterior oblique caudal view, arrow in **A**), proximal-mid LAD DES implantation (anteroposterior cranial view, arrow in **B**), and TIMI 3 flow

A posterior STEMI Killip class IV was confirmed at our hospital. We immediately performed coronary angiography, which revealed critical stenosis of 98% in the proximal LCx, significant stenosis of 80% in the proximal LAD, and a normal RCA (Fig. 8). Complete revascularization was performed with PCI in the LCx and LAD, which resulted in TIMI 3 flow and ST-segment resolution on ECG (Fig. 9).

**Fig. 10:**
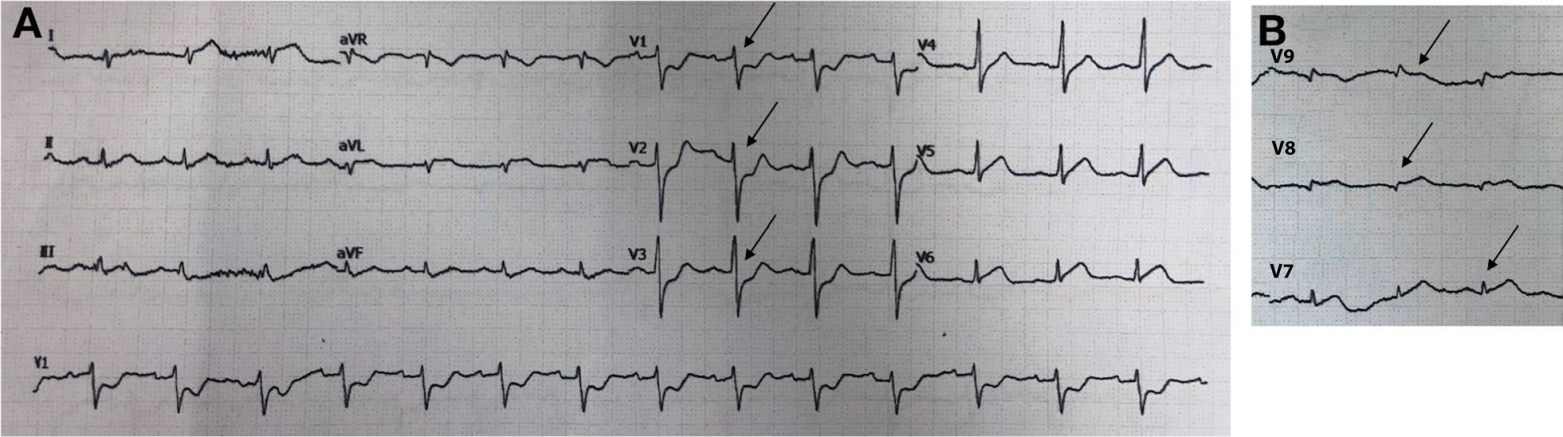
The ECG of the referring hospital. **A** Arrows display anteroseptal ST-segment depression in V1–V3. **B** Arrows display posterior ST elevation in V7–V9

**Fig. 11:**
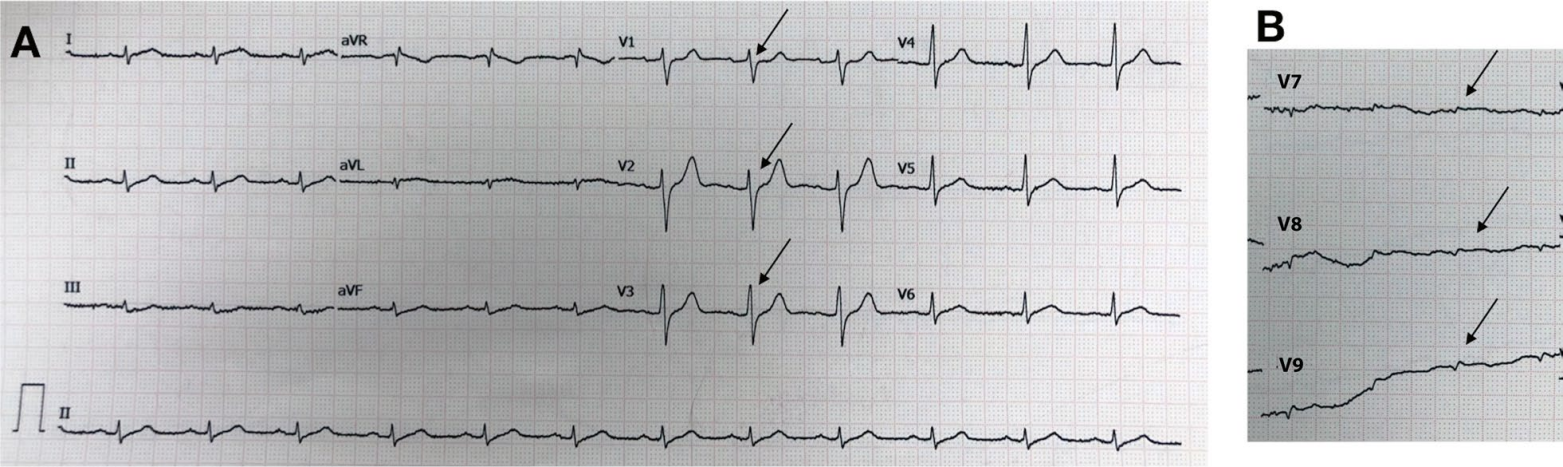
The ECG that was performed upon arrival at the PPCI hospital. **A** Arrows display isoelectric ST-segment anteroseptal in V1–V3. **B** Arrows display persistent ST-segment elevation in V7–V9

The third case included a 53-year-old Asian man who presented with symptoms of chest pain radiating to his back for the previous 10 hours, accompanied by cold sweat, nausea, and vomiting. He had a history of smoking but no hypertension, diabetes, or previous heart disease. His vital signs at our hospital were as follows: blood pressure, 120/80 mmHg; pulse rate, 85 beats per minute; respiration, 20 breaths per minute; and peripheral capillary oxygen saturation, 98%. Chest examination showed no abnormalities. At the referring hospital, ECG revealed ST depression in V1–V3 and ST elevation in V8–V9 (Fig. 10). However, ECG at our hospital demonstrated isoelectric ST segments in the anteroseptal leads (V1–V3) with persistent ST elevation in the posterior leads (Fig. 11). Additionally, the troponin T value increased to 13,061 ng/L. Echocardiography revealed a slightly hypokinetic posterior wall with a left ventricular ejection fraction of 58%.

**Fig. 12:**
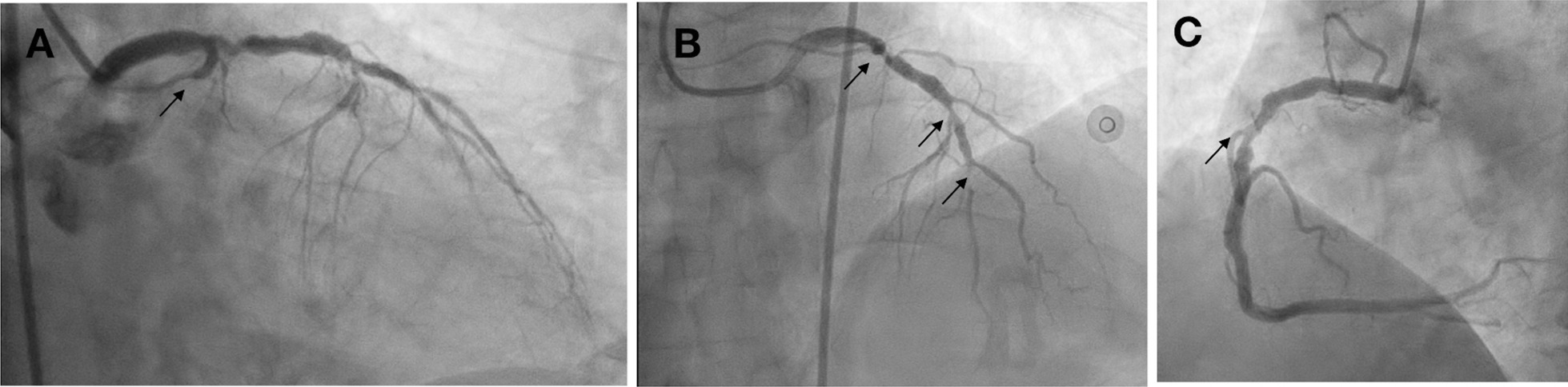
Coronary angiography showing total occlusion of the proximal LCx (right anterior oblique caudal view, arrow in **A**), significant stenosis (70%) of the proximal LAD, subtotal stenosis (98%) of the mid-LAD (anteroposterior cranial view, arrows in **B**), and nonsignificant stenosis (50%) of the mid-RCA (left anterior oblique view, arrow in **C**)

**Fig. 13:**
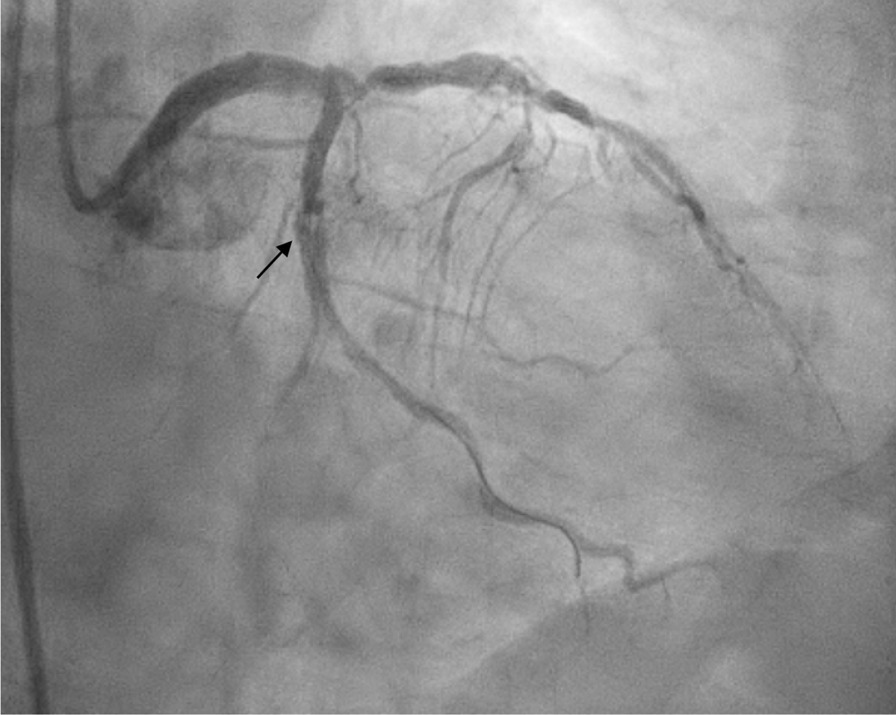
Right anterior oblique caudal view of left coronary angiogram after DES implantation in the proximal–distal LCx, TIMI 3 flow (arrow)

A posterior STEMI Killip class I was confirmed. Coronary angiography revealed total occlusion of the proximal LCx, significant stenosis (70%) of the proximal LAD, which was accompanied by subtotal stenosis (98%) of the mid LAD, and nonsignificant stenosis (50%) of the mid-RCA (Fig. 12). Stents were placed in the proximal–distal LCx, resulting in TIMI 3 flow and resolution of the ST-segment elevations (Fig. 13). Residual coronary disease in the LAD was stented electively during staged procedures*.*

The patients were kept under observation in the intensive care unit after PPCI for 1–2 days and were discharged after 3–6 days of treatment. We do not have post-discharge follow-up data because the patients received follow-up at the referring hospital. However, we remain curious whether there was an improvement of symptoms or Ejection Fraction (EF) recovery in the patients.

## Discussion

On the basis of the European Society of Cardiology congress of 2017 guidelines, the ECG criterion of IPMI includes an ST elevation of more than 0.5 mm in the posterior leads V7–V9; IPMI is strongly suspected if ST-segment depression in leads V1–V3 is observed, especially when accompanied by positive T waves. [9] However, ischemia does not always appear in V1–V3 during IPMI. A study conducted by Aqel *et al*. using a model of temporary balloon occlusion to produce ischemia proved that 11% of patients with LCx occlusion had ST elevations in the posterior lead, with no ST elevation or depression being observed in any other leads [10]. Another recent study by Vogiatzis *et al*. showed that, among 186 patients with ACS, 28 patients (15.1%) required a posterior ECG to diagnose STEMI before reperfusion therapy, which indicated that these were cases of IPMI. Among these patients, 11 had no indicative abnormalities on a 12-lead ECG. This circumstance may lead to the misdiagnosis of IPMI, thus resulting in delayed revascularization in the previously reported 11 patients if the recording of the posterior ECG has not been done accordingly. Vogiatzis *et al*. also performed a multivariate regression analysis showing that 15-lead ECG was the sole factor that was significantly associated with MI diagnosis. In contrast, ST-segment depression in V1–V4 did not show statistically significant results [11]. Table 1 reviews some of the previous studies concerning the importance of 15-lead ECG in IPMI cases. Indeed, IPMI has been widely discussed in previous studies, including the importance of posterior lead ECG [6, 11–16]. A recent study in 2021 by Shimojo *et al*. suggested that the V7–V9 lead helps diagnose IPMI and might improve the prognosis [16]. However, IPMI is still a rare occurrence that remains underdiagnosed [15]. Therefore, we believe that it is crucial to reemphasize the importance of routine 15-lead ECGs in allowing for the early recognition of IPMI.

**Table 1:** Review of the previous studies of isolated posterior myocardial infarct

Number	Title	Author	Number of subject	Results
1.	Acute myocardial infarction (AMI) with isolated ST-segment elevation in posterior chest leads V7–V9	Matezky *et al*. 1999 [12]	33 patients	Study subjects included 33 consecutive patients with ischemic chest pain suggestive of AMI who did not have ST elevation on standard ECG but had ST elevation in V7–V9 Among the patients, ST depression was noted in leads V1–V3 in 20 patients (61%), ECG pathologic Q waves were noted in leads V7–V9 in 26 patients (79%), and LCx artery was the infarct-related artery in all the catheterized patients (20 patients)
2.	Prevalence and outcome of ST-segment elevation in posterior electrocardiographic leads during myocardial infarction	Oraii *et al*. 1999 [6]	7 patients	Among 210 consecutive patients with AMI, 19 patients (9%) had STE of > 1 mm in two or more posterior leads, either as an isolated finding [7 cases (3.3%)] or in association with STE at the inferior or lateral sites [12 cases (5.7%)] In-hospital complications were significantly more frequent in the IPMI group compared with matched controls [Mantel–Haenszel odd ratio (OR) 7, confidence interval (CI) 1.28–28.43]
3.	Importance of posterior chest leads in patients with suspected myocardial infarction, however, nondiagnostic, routine 12-lead electrogram	Agarwal *et al*. 1999 [13]	18 patients	Among 58 patients who had clinically suspected MI with the use of nondiagnostic routine 12-lead ECG, 18 patients had ST elevations of > 0.1 mV or Q waves in ≥ 2 posterior leads Door-to-balloon times (107 minutes versus 72 minutes; *p* < 0.01) were longer among patients with IPMI, as fewer patients received reperfusion within 90 minutes (30% versus 71%; *p* < 0.01)
4.	Clinical characteristics and reperfusion times among patients with an isolated posterior myocardial infarction	Waldo *et al*. 2013 [14]	20 patients	On the basis of the registry between 2008 and 2012, among 318 patients who underwent revascularization for STEMI, 20 patients (6%) had an isolated posterior MI
5.	The importance of the 15-lead versus 12-lead ECG recordings in the diagnosis and treatment of right ventricle and left ventricle posterior and lateral wall AMI	Vogiatzis *et al*. 2014 [11]	28 patients	Among 186 patients with acute coronary syndrome, posterior lead ECG was required in 28 patients (15.1%) to establish a STEMI diagnosis for the performance of reperfusion therapy A multivariate regression analysis showed that 15-lead ECG was the sole factor that was significantly associated with MI diagnosis (OR 2.43, *p* = 0.04)
6.	Reperfusion times and in-hospital outcomes among patients with an isolated posterior myocardial infarction: insights from the National Cardiovascular Data Registry	Waldo *et al*. 2014 [15]	824 patients	Among 117,739 subjects with STEMI, 824 patients (0.7%) were diagnosed with an isolated PMI In a subject with IPMI, fewer patients achieved a door-to-balloon time of less than 90 minutes (83% versus 89%, *p* < 0.01).
7.	Prevalence and prognosis of isolated posterior ST-segment elevation acute myocardial infarction using synthesized-V7–9 lead	Shimojo *et al*. 2021 [16]	10 patients	Among 142 consecutive patients with STEMI with the culprit lesion on LCx, 10 patients (7.0%) had ST elevation only in synthesized V7–V9 lead who classified as STEMI-LCx-synV7–V9 group and the remaining as STEMI-LCx-12ECG group [132 patients (93%)] The patients with STEMI-LCx-synV7–V9 had significantly higher incidences of cardiac death within 3 months and 1 year (30.0% versus 6.1%, *p* = 0.031, 30.0% versus 7.6%, *p* = 0.050, respectively) and mechanical complications in each follow-up period (20.0% versus 1.5 %, *p* = 0.025) than those with STEMI-LCx-12ECG

Our cases showed transient reciprocal features in the anterior lead due to the evolutionary course of ST elevation, which ultimately returned to baseline [17, 18]. Another possibility that may contribute to temporary anterior ECG changes is the concomitant significance to critical LAD stenosis, which was found in all our cases, although we did not find any studies or reports that have analyzed the correlation of IPMI with this particular condition.

In our second case, a V7–V9 lead examination was not performed at the referring hospital. As a result, the diagnosis of IPMI was made at our hospital, resulting in delayed therapy with a door-to-balloon time of more than 90 minutes. A study conducted in 2013 (based on national registry results) showed that the mean time from the initial ECG to PPCI was significantly longer among subjects with IPMI (69 minutes versus 61 minutes, *p* < 0.01), and fewer patients had a door-to-balloon time that was less than 90 minutes (83% versus 89%, *p* < 0.01) [15]. A delayed reperfusion time in STEMI leads to delayed treatment, thus resulting in more severe myocardial infarctions and a higher mortality rate. A previous study showed that every 30 minutes of delay was associated with a relative risk for 1-year mortality of 1.075 (95% CI 1.008–1.15, *p* = 0041) [19]. The posterior leads for acute MI detection demonstrated a sensitivity of 60%, a specificity of 89%, a positive predictive value of 91%, and a negative predictive value of 55% [11].

Owing to a lack of knowledge, a normal 12-lead ECG even in symptomatic patients does not always trigger the physician to perform an ECG with additional posterior leads. The use of the 15-lead ECG (as described in our cases) in symptomatic patients with a high suspicion of ACS will decrease missed diagnoses of IPMI and/or delays in reperfusion therapy (either thrombolytic or PPCI procedures), especially in cases with normal ECGs or temporary changes in the precordial lead. However, the placement of additional leads should be performed by experienced personnel so that the results are obtained faster and false interpretations of the ECG can be avoided. Furthermore, the use of a 15-lead ECG has a financial impact and increases costs, as it requires additional resources and personnel training. In our opinion, the additional training requirements and costs will not be difficult to meet, as it is only necessary to place the additional three leads in the correct posterior positions. This method will significantly contribute to saving the lives of patients and achieving early revascularization in IPMI. Future studies with larger subject groups (such as with the use of large national databases) will be needed to determine the significance of 15-lead ECG in diagnosing IPMI in patients with a strong suspicion of ACS.

## Conclusion

We describe three cases of isolated posterior MI that were detected only after the addition of posterior leads (V7–V9) to the standard 12-lead ECG. Although rare, isolated posterior MI remains underdiagnosed and is often treated at a later stage, which is known to be associated with worse outcomes in acute coronary syndromes. We advocate the routine use of a 15-lead ECG in all symptomatic patients in whom a 12-lead ECG is not sufficiently informative to make a proper diagnosis of IPMI.

## Data Availability

Not applicable.

## Abbreviations

IPMI: Isolated posterior myocardial infarction
ECG: Electrocardiography
LCx: Left circumflex coronary artery
ACS: Acute coronary syndrome
PPCI: Primary percutaneous coronary intervention
STEMI: ST elevation myocardial infarction
MI: Myocardial infarction
RCA: Right coronary artery
LAD: Left anterior descending artery
NSTEMI: Non-ST-elevation myocardial infarct
VF: Ventricular fibrillation
OM1: Obtuse marginal 1
DES: Drug-eluting stent

## References

[1] Rich MW, Imburgia M, King TR, Fischer KC, Kovach KL. Electrocardiographic diagnosis of remote posterior wall myocardial infarction using unipolar posterior lead V9. Chest. 1989;96(3):489–93. 10.1378/chest.96.3.489. 2788559

[2] Van Gorselen EOF, Verheugt FWA, Meursing BTJ, Oude Ophuis AJM. Posterior myocardial infarction: the dark side of the moon. Neth Heart J. 2007;15(1):16–21. PMC1847720 17612703

[3] Perloff JK. The recognition of strictly posterior myocardial infarction by conventional scalar electrocardiography. Circulation. 1964;30(5):706–18. 10.1161/01.CIR.30.5.706. 14226169

[4] Levis J. ECG diagnosis: isolated posterior wall myocardial infarction. Perm J. 2015;19(4):e143–4. 10.7812/TPP/14-244. PMC5293126 26828074

[5] Thejanandan Reddy CS, Rajasekhar D, Vanajakshamma V. Electrocardiographic localization of infarct related coronary artery in acute ST elevation myocardial infarction. J Clin Sci Res. 2013;2:151–60.

[6] Oraii S, Maleki M, Abbas Tavakolian A, *et al*. Prevalence and outcome of ST-segment elevation in posterior electrocardiographic leads during acute myocardial infarction. J Electrocardiol. 1999;32(3):275–8. 10.1016/S0022-0736(99)90110-3. 10465571

[7] Brady WJ, Erling B, Pollack M, Chan TC. Electrocardiographic manifestations: acute posterior wall myocardial infarction. J Emerg Med. 2001;20(4):391–401. 10.1016/s0736-4679(01)00318-3. 11348821

[8] Brown L, Sims J, Conforto A. Posterior myocardial infarction with isolated ST elevations in V8 and V9: is this an “ST elevation MI”? CJEM. 2003;5(2):115–8. 10.1017/s1481803500008265. 17475102

[9] Ibanez B, James S, Agewall S, Antunes MJ, Bucciarelli-Ducci C, Bueno H, Caforio ALP, Crea F, Goudevenos JA, Halvorsen S, Hindricks G, Kastrati A, Lenzen MJ, Prescott E, Roffi M, Valgimigli M, Varenhorst C, Vranckx P, Widimsky P, ESC Scientific Document Group. 2017 ESC Guidelines for the management of acute myocardial infarction in patients presenting with ST-segment elevation: the Task Force for the management of acute myocardial infarction in patients presenting with ST- segment elevation of the European Society of Cardiology (ESC). Eur Heart J. 2018;39(2):119–77. 10.1093/eurheartj/ehx393.

[10] Aqel RA, Hage FG, Elipeddi P, Blackmon L, McElderry HT, Kay N, Plumb V, Iskandrian AE. Usefulness of three posterior chest leads for the detection of posterior wall acute myocardial infarction. Am J Cardiol. 2009;103(2):159–64. 10.1016/j.amjcard.2008.09.008. 19121429

[11] Vogiatzis I, Koulouris E, Ioannidis A, Sdogkos E, Pliatsika M, Roditis P, Goumenakis M. The importance of the 15-lead versus 12-lead ECG Recordings in the diagnosis and treatment of right ventricle and left ventricle posterior and lateral wall acute myocardial infarctions. Acta Inform Med. 2019;27(1):35–9. 10.5455/aim.2019.27.35-39. PMC6511271 31213741

[12] Matezky S, Freimark D, Feinberg MS, Novikov I, Rath S, Rabinowitz B, Kaplinsky E, Hod H. Acute myocardial infarction with isolated ST-segment elevation in posterior chest leads V7–9. J Am Coll Cardiol. 1999;24(3):748–53. 10.1016/s0735-1097(99)00249-1 10483956

[13] Agarwal JB, Khaw K, Aurignac F, LoCurto A. Importance of posterior chest leads in patients with suspected myocardial infarction, however, nondiagnostic, routine 12-lead electrocardiogram. Am J Cardiol. 1999;83:323–6. 10.1016/s0002-9149(98)00861-3 10072216

[14] Waldo SW, Armstrong EJ, Kulkarni A, Hoffmayer KS, Hsue P, Ganz P, McCabe JM. Clinical characteristics and reperfusion times among patients with an isolated posterior myocardial infarction. J Invasive Cardiol. 2013;25(8):371–5. 23913600

[15] Waldo SW, Brenner DA, Li S, Alexander K, Ganz P. Reperfusion times and in hospital outcomes among patients with an isolated posterior myocardial infarction: insight from the National Cardiovascular Data Registry (NCDR). Am Heart J. 2014;167(3):350–4. 10.1016/j.ahj.2013.11.011. 24576519

[16] Shimojo K, Takagi K, Morita Y, Kanzaki Y, Nagai H, Watanabe N, Yoshioka N, Yamauchi R, Komeyama S, Sugiyama H, Imaoka T, Sakamoto G, Ohi T, Goto H, Tsuboi H, Morishima I. Prevalence and prognosis of isolated posterior ST-segment elevation acute myocardial infarction using synthesized-V7–9 lead. Cardiovasc Interv Ther. 2021. 10.1007/s12928-021-00796-1. 34542792

[17] Brady WJ, Hwang V, Sullivan R, Chang N, Beagle C, Carter CT, Martin ML, Aufderheide TP. A comparison of 12- and 15-lead ECGS in ED chest pain patients: impact on diagnosis, therapy, and disposition. Am J Emerg Med. 2000;18(3):239–43. 10.1016/S0735-6757(00)90112-8. 10830674

[18] Wong C. Usefulness of leads V7, V8, and V9 ST elevation to diagnose isolated posterior myocardial infarction. Int J Cardiol. 2011;146(3):467–9. 10.1016/j.ijcard.2010.10.137. 21131078

[19] De Luca G, Suryapranata H, Ottervager JP, Antman EM. Time delay to treatment and mortality in primary angioplasty for acute myocardial infarction every minute of delay counts. Circulation. 2004;109(10):1223–5. 10.1161/01.cir.0000121424.76486.20. 15007008

